# Supplementary material for: Validating and automating learning of cardiometabolic polygenic risk scores from direct-to-consumer genetic and phenotypic data: implications for scaling precision health research
Source: Hum Genomics. 2022 Sep 8;16:37. doi: 10.1186/s40246-022-00406-y (PMC9452874; doi:10.1186/s40246-022-00406-y)

# Supplementary Material B

Number of variants per genotyping platform pre- and pos-QC, and final genotyping call rate.


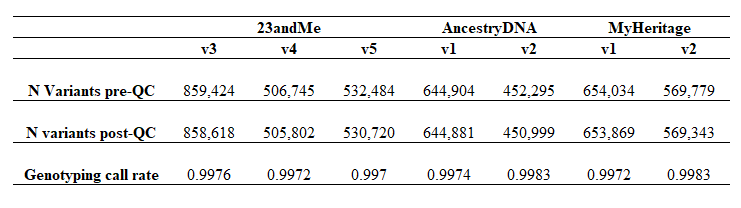

Supplement: Supplementary file 2 — Additional file 2. Number of variants and Genotyping Call Rate. [file 40246_2022_406_MOESM2_ESM.docx]
